# TcTASV-C, a Protein Family in *Trypanosoma cruzi* that Is Predominantly Trypomastigote-Stage Specific and Secreted to the Medium

**DOI:** 10.1371/journal.pone.0071192

**Published:** 2013-07-29

**Authors:** Guillermo Bernabó, Gabriela Levy, María Ziliani, Lucas D. Caeiro, Daniel O. Sánchez, Valeria Tekiel

**Affiliations:** Instituto de Investigaciones Biotecnológicas – Instituto Tecnológico de Chascomus (IIB-INTECH), Universidad Nacional de San Martín (UNSAM) – Consejo Nacional de Investigaciones Científicas y Técnicas (CONICET), Buenos Aires, Argentina; University of Melbourne, Australia

## Abstract

Among the several multigene families codified by the genome of *T. cruzi*, the TcTASV family was the latest discovered. The TcTASV (Trypomastigote, Alanine, Serine, Valine) family is composed of ∼40 members, with conserved carboxi- and amino-*termini* but with a variable central core. According to the length and sequence of the central region the family is split into 3 subfamilies. The TcTASV family is conserved in the genomes of – at least – lineages TcI and TcVI and has no orthologues in other trypanosomatids. In the present work we focus on the study of the TcTASV-C subfamily, composed by 16 genes in the CL Brener strain. We determined that TcTASV-C is preferentially expressed in trypomastigotes, but it is not a major component of the parasite. Both immunoflourescence and flow cytometry experiments indicated that TcTASV-C has a clonal expression, *i.e.* it is not expressed by all the parasites of a certain population at the same time. We also determined that TcTASV-C is phosphorylated and glycosylated. TASV-C is attached to the parasite surface by a GPI anchor and is shed spontaneously into the medium. About 30% of sera from infected hosts reacted with TcTASV-C, confirming its exposition to the immune system. Its superficial localization and secretory nature suggest a possible role in host-parasite interactions.

## Introduction

*Trypanosma cruzi* is the hemoflagellate parasite that causes Chagaśdisease, also known as American Trypanosomiasis. Thirty–40% of infected patients will develop a determinate form of chronic disease (*i.e.,* cardiac, digestive (megaoesophagus and mega colon), or cardiodigestive). The symptoms appear generally only 20–40 years after the initial infection, when treatment is poorly effective. [1]. Although several studies indicate that there would be a correlation between *T. cruzi* lineage and clinical symptoms, no proven associations are evident at present and both the parasite and host genotypes are important in determining the tissue distribution, physiopathology and eventual outcome of *T. cruzi* infection [1]–[4]. Regardeless the clinical form, there is a consensus that the pathology is caused by immunological imbalances that are triggered by the parasite's antigens [5], [6]. The disease is transmitted mostly when the parasite is in the trypomastigote stage. In the case of vectorial transmission, the transmission is caused by metacyclic trypomastigotes. If the infection is acquired congenitally or through transfusions, the transmission occurs by circulating trypomastigotes. Once inside the vertebrate host, the trypomastigote must invade a nucleate cell, where it differentiates to the amastigote stage and multiplies by binary fission in the cytoplasm. After several rounds of division, amastigotes differentiate again into trypomastigotes and the cell is lysed. The trypomastigotes are released to blood and spread the infection into the different organs/tissues, where trypomastigotes invade other host cells, to start again the multiplication cycle [7]. During the first months after primoinfection, circulating trypomastigotes are easily found in blood and, if the disease is diagnosed, the treatment is effective. The drugs that are currently available to treat Chagas' disease have serious side effects therefore, genes expressed differentially in trypomastigotes are promising targets for drug or vaccine development [1].

The completion of the sequencing of the genome of *T. cruzi* has given an insight into the parasite genome, which has 3700 species-specific genes. Several protein families have been identified previously (trans-sialidase (TS), mucin, gp63, gp82/85, amastin, DGF-1) or as a consequence (mucin-associated surface proteins, MASP) of the sequencing of the *T. cruzi* genome [8]–[19] [20], [21]. While some of those gene families are expressed throughout the parasite's life cycle, others have differential expression at a certain stage. Many of the genes expressed in trypomastigotes have been associated with recognition, adhesion and/or active cell invasion or escape of the immune response [22]–[33].

We have recently identified a novel family of predicted surface proteins that was named TcTASV, due to the fact that it was first noticed from a trypomastigote cDNA library and has a biased composition in alanine, serine and valine [34]. In the CL Brener strain –the first sequenced *T. cruzi* genome and the most extensively annotated up to date- we found 41 TcTASV genes. In other *T. cruzi* strains (RA, lineage VI and Dm28, lineage I) we experimentally found a similar number of TcTASV genes [34]; the family is also present in the recently sequenced Sylvio strain [35]. Interestingly, despite its broad and conserved presence in *T. cruzi* strains, TcTASV has no orthologs in other trypanosomatids.

TcTASV genes have highly conserved 3′UTRs, and both the amino- and carboxi-*termini* of the gene products (85–100% amino acid identity). The family is split into 3 main subfamilies (A, B and C) according to the length and composition of the central region, which is variable [34]. Almost all TcTASV gene products have a predicted signal peptide and a signal for GPI anchoring, thus suggesting that this family can be located at the parasite surface and/or be secreted to the milieu. Bioinformatic algorithms also predicted that TcTASVs members are phosphorylated and highly glycosylated [34].

The TcTASV-A subfamily is composed by 21 genes in the CL Brener strain and its expression as a ∼18 kDa polypeptide in trypomastigotes has been demonstrated in our previous work. Peptides from 5 TcTASV-A genes were also recently identified in the trypomastigote proteome [36]. The TcTASV-B subfamily is composed only by 4 members in CL Brener and no genes were detected in the Dm28 (lineage I) strain. The TcTASV-C subfamily -composed by 16 members in the CL Brener strain, 20 in RA and 15 in Dm28- is the subfamily predicted to be more thickly glycosylated. One member has been identified as a potential vaccine candidate [37], but there is no data regarding its expression. All this background information prompted us to characterize the TcTASV-C subfamily. Briefly, we have determined that TcTASV-C is expressed mainly in the trypomastigote stage as a ∼60 kDa protein, whose carbohydrates are responsible for at least 10 kDa of the relative molecular mass. TcTASV-C is clonally expressed and it is not a major component of the parasite surface. Moreover, about 30% of sera from infected hosts recognized TcTASV-C, giving evidence of its expression during the course of the natural infection and its contact with the immune system of the host.

## Materials and Methods

### Ethics Statement

All experiments using animals were approved by the Animal Ethical Committee of our Institution (CICUAE, Universidad Nacional de San Martín) and were carried out in accordance with national and international welfare grounds.

### Parasites and antigen preparation

The parasites stocks used were CL Brener (TcVI), RA (TcVI) and Sylvio ×10/7 (TcI). CL Brener and RA strains were gifts of Dr. B. Zingales and Dr. S.M. González Cappa, respectively, to our Institution [38], [39]. Typing of *T. cruzi* evolutionary lineages was carried out by PCR (results not shown) [40]. For *in vitro* assays, parasites were obtained from axenic cultures (epimastigotes and metacyclic trypomastigotes) or by infection of Vero cell monolayers (amastigotes and released trypomastigotes) as previously described [34]. As a rule, *T. cruzi* stocks are kept in liquid nitrogen and all strains are regularly thawed once a year to preserve the strain's characteristics.

Essentially pure parasites of each stage were used (less than 5% of other stages). Parasites were washed with PBS and –otherwise indicated- lysed by incubation in a RIPA-like buffer (50 mM Tris pH = 8, 150 mM NaCl, 1 mM Cl_2_Mg, 0.1% SDS, 1% NP-40, 1 mM EDTA, 1 mM DTT) plus protease inhibitor cocktail (Sigma) and DNAseI (10 µg/ml) for 30 min on ice and clarified by centrifugation. Proteins were quantified by Bradford and the concentration was adjusted to 1 µg/µl. Parasite lysates were stored –aliquoted- at −80°C until use.

### Cloning, expression and purification of TASV-C

**Figure 1:**
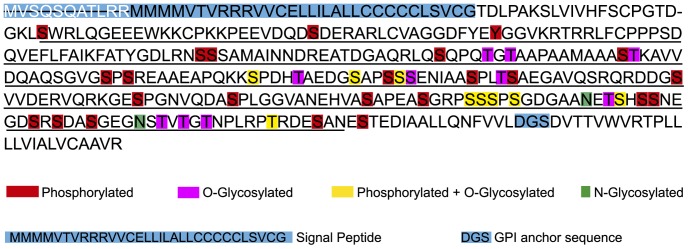
Sequence of the protein product of *Tcruzi_1863-4-1211-93*, a representative member of TcTASV-C subfamily. The internal region that was cloned to produce the recombinant protein TcTASV-C_GST_ is underlined (amino acids 65 to 330). Amino acids that are predicted to have post-translational modifications are highlighted. The signal peptide that is present in the N-terminal region and the consensus sequence for the addition of a GPI anchor in the C-terminal region are both highlighted in blue (black letters). The first amino acids (white letters, highlighted in blue) correspond to a wrong-predicted amino-terminal region.

For cloning and expression of one TcTASV-C gene, nucleotides 1021 to 223 (amino acids 65 to 330) of the predicted ORF *Tcruzi_1863-4-1211-93* were amplified by PCR from the clone G53E20 (GenBank Acc AZ050960) using Pfu DNA polymerase and the primers CDS_int_L (ggatcctgagttggcgtcttcaag) and CDS_int_R (tttgcactttcgtctctg). The products were cloned into the pGEM-T Easy vector and sequenced. This internal region of TcTASV-C was subcloned into the pGEX-3X vector (Pharmacia) in frame with glutathione-S-tranferase (GST) to obtain the construct TcTASV-C_GST_ (Fig. 1). TcTASV-C_GST_ was expressed as a recombinant protein in *E. coli* BL21 and purified by standard methodology [41].

### Antisera development and IgG purification

Recombinant TcTASV-C_GST_ was used to produce anti-TcTASV-C serum in mice. Briefly, mice were immunized by injection of 10 µg of TcTASV-C_GST_ emulsified in complete Freund's adjuvant (1^st^ dose) and boosted with 5 µg of TcTASV-C_GST_ in incomplete Freund's adjuvant (2^nd^ and 3^rd^ doses) [42]. The anti-TcTASV serum was depleted of anti-GST antibodies by incubation with a Glutathione Sepharose 4 Fast Flow resin (Pharmacia Biotech) coupled with 1 mg of pure GST in the presence of protease inhibitors. The total IgG fraction of the serum was then purified with protein G columns (HiTrap, GE Healthcare Life Sciences). Finally, specific anti- TcTASV-C antibodies were affinity-purified by a column coupled with the recombinant protein (SulfoLink^®^ Kits; Thermo Scientific) following the manufacturer's instructions. The specificity of the anti-TcTASV-C antibodies was established by competition assays (File S1). Antibodies were used at 0.1 µg/ml for western blot and at 10 µg/ml for flow cytometry and immunofluorescence assays.

### Western blot

Approximately the equivalent of 20×10^6^ trypomastigotes, 14×10^6^ epimastigotes, or 55×10^6^ amastigotes (unless otherwise indicated) were loaded in each gel lane to assure similar protein content among the different parasite-stages. Parasite proteins (∼20 µg/lane) were electrophoresed on 10% denaturing polyacrylamide gels, and transferred to nitrocellulose membranes by standard methodologies. The membranes were blocked with PBS –3% non-fat milk, and incubated with purified anti-TcTASV-C antibodies (O.N., 4°C), rabbit anti-GDH antisera (1∶6000, 1 h, R.T.) or rabbit anti-TcSR62 (1∶1000, 1 h, R.T.) [43], [44]. Peroxidase-labeled goat anti-mouse or goat anti-rabbit (both from Thermo Scientific) were used as secondary antibodies. SuperSignal West Femto (when indicated) and SuperSignal West Pico (both from Thermo Scientific) were used as chemiluminescent substrates to develop TcTASV-C and loading controls, respectively.

### Enzymatic treatment of trypanosome extracts

For phosphatidylinositol-specific phospholipase C (PI-PLC) treatment, cell-derived trypomastigotes were washed twice in PBS and resuspended to a concentration of 10^8^ parasites/ml in 500 µl of serum-free DMEM (Life Technologies). Parasites were incubated for 1 h at 37°C with or without the addition of 2 U of PI-PLC from *Bacillus cereus* (Sigma Aldrich). Control was performed by incubation 5×10^7^ parasites in serum-free medium at 0°C. The medium containing the secreted/released antigens and the pellet containing parasites were separated by centrifugation at 4000 g for 10 min at 4°C. Secreted/released antigens and pelleted parasites (washed twice with PBS) were processed by western blot as described above.

The equivalent of 25×10^6^ trypomastigotes -lysed by freeze and thaw cycles- were deglycosylated under denaturing conditions in the presence of Triton X-100 (0.75%) at 37°C for 5 h with E-DEGLY kit (Sigma), following the manufacturer protocol. The E-DEGLY kit includes enzymes to remove N-linked and O-linked carbohydrates from glycoproteins (PNGaseF, Endo-O-Glycosidase, α-2(3,6,8,9)-Neuraminidase [Sialidase A], β-1,4-Galactosidase and β-N-Acetylglucosaminidase).

For dephosphorylation assays, 25×10^6^ trypomastigotes were lysed for 30 min at 4°C in a 50 mM Tris buffer (pH = 8.8) containing 1.5 mM MgCl_2_, 1% TritonX-100, 1 mM DTT and protease inhibitors, and then incubated with 15 U of calf intestinal alkaline phosphatase (CIAP, Promega) or mock-treated for 1 h at 37°C. The levels of deglycosylation and dephosphorylation were analyzed by western blot using anti-TcTASV-C antibodies.

### Surface labeling and detection of parasites

For immunofluorescence assays, parasites resuspended at 5×10^6^/ml in PBS were layered onto 10-mm glass cover slides pretreated with polylysine (Sigma) and fixed in 4% paraformaldehyde (PFA) in PBS. Cover slides were saturated in blocking buffer (3% goat serum, 2% BSA in PBS) for 1 h, washed twice in PBS, and incubated with either anti-TcTASV-C antibodies or IgG purified from sera of control mice. The cover slides were washed three times and incubated with Alexa-Fluor 488-conjugated immunoglobulins (Molecular Probes) for 1 h at room temperature. After the incubation with the secondary antibody, parasites were washed, incubated with saponin (0.5%; 10 min), washed again, and incubated either with DAPI or propidium Iodide (50 µg/ml; 15 min) for DNA stain. Finally, cover slides were mounted in antifade reagent (FluorSave, Calbiochem), observed under a microscope (Nikon E600) using appropriate fluorescence emission filters, and photographed.

Labeling of parasites for flow cytometry was performed essentially as described by Vitelli-Avelar *et al*., with slight modifications [45]. In short, live trypomastigotes (10^6^/assay) were incubated for 1 h at 4°C with anti-TcTASV-C or control antibodies in PBS 10% bovine fetal serum. After two washes, parasites were incubated with Alexa-Fluor 488-conjugated goat anti-mouse IgG (Molecular Probes, 1∶1000 in PBS, 10% BFS) for 1 h on ice. Following two more washes, parasites were fixed with FACS buffer (1% PFA, 0.01% sodium azide in PBS) and stored at 4°C in the dark [46]. Samples were acquired on a FACSCalibur (Becton Dickinson), and data were analyzed with WinMDI 2.8 software. All experiments were carried out at least twice.

### Screening of sera from infected hosts

Twenty-eight sera from *T. cruzi* infected rabbits and 12 non-infected controls, from the lab's stock were used. All the sera were tested against *T. cruzi* antigens before screening the reactivity anti-TcTASV-C. Sera proceeded from rabbits infected with RA (n = 3), K-98 (n = 2), CA-I (n = 2), Y (n = 2), UP (n = 3), Awp (n = 3) and Tul0 (n = 2) *T. cruzi* strains [47]. The infecting strains of 11 sera were unknown.

The reactivity of sera from infected hosts was analyzed by ELISA. Briefly, 96 wells-plates were coated with 100 ng of recombinant TcTASV-C_GST_ or GST per well. Sera were assayed at 1∶50, 1∶100 and 1∶200 dilutions and peroxidase-coupled secondary antibodies were used at 1∶10000 (goat anti-human or goat anti-rabbit, both from Thermo Scientific). Color development was carried out employing TMB (BD Biosciences); the reaction was stopped with H_2_SO_4_ 2N and the optical density registered at 450 nm (OD_450_) (Benchmark, Microplate Reader, BioRad). Reactivity against TcTASV-C was expressed as the ratio of the OD_450_ for TASV-C and GST for a certain serum (OD_450_ TcTASV-C/OD_450_ GST). Sera were considered positive when the ratio was higher than the cut-off value, calculated as the media of ratios of the non-infected sera plus 2 standard deviations (SD).

### Bioinformatic predictions

The programs NetPhos, NetOGlyc and NetNGlyc available at the server of the Center for Biological Sequence Analysis (CBS; http://www.cbs.dtu.dk/services/), were used to predict post- translational modifications of proteins.

### Statistical analysis

All graphics and statistical analyses were performed using the GraphPad Prism 4.0 software. Comparison between two groups was carried out with the Student t test.

## Results

TcTASV-C is the TcTASV subfamily whose gene products are proteins predicted to have 330-360 amino acid length. We have previously identified TcTASV-C genes in CL Brener, RA and Dm28 strains [34]. *A posteriori*, we also identified *in silico* the TcTASV-C subfamily in the genomes of the Sylvio ×10/1 and JR cl4 strains [35].

To study the molecular and antigenic profile of the TcTASV-C subfamily, we selected a region spanning from Ser65 to Asn330 of *Tcruzi_1863-4-1211-93* (Fig. 1, underlined region). This region was cloned fused to GST, expressed as recombinant protein in *E. coli* and purified by affinity chromatography to glutathion agarose. *Tcruzi_1863-4-1211-93* is still annotated as an ORF at the TriTryp database (http://TritrypDB.org), probably because it remains as a scaffold of 1292 bp that has not been associated with any chromosome yet (File S2) [48]. However, we had previous evidence of its mRNA expression in trypomastigotes by northern blot, which suggests that it is in fact a gene [34]. Moreover, we have chosen *Tcruzi_1863-4-1211-93* to work with because it has 100% identity with FN599132.1, another TASV-C gene that was identified in the RA strain.

### TcTASV-C is expressed as a ∼60kDa protein, mainly in trypomastigotes

**Figure 2:**
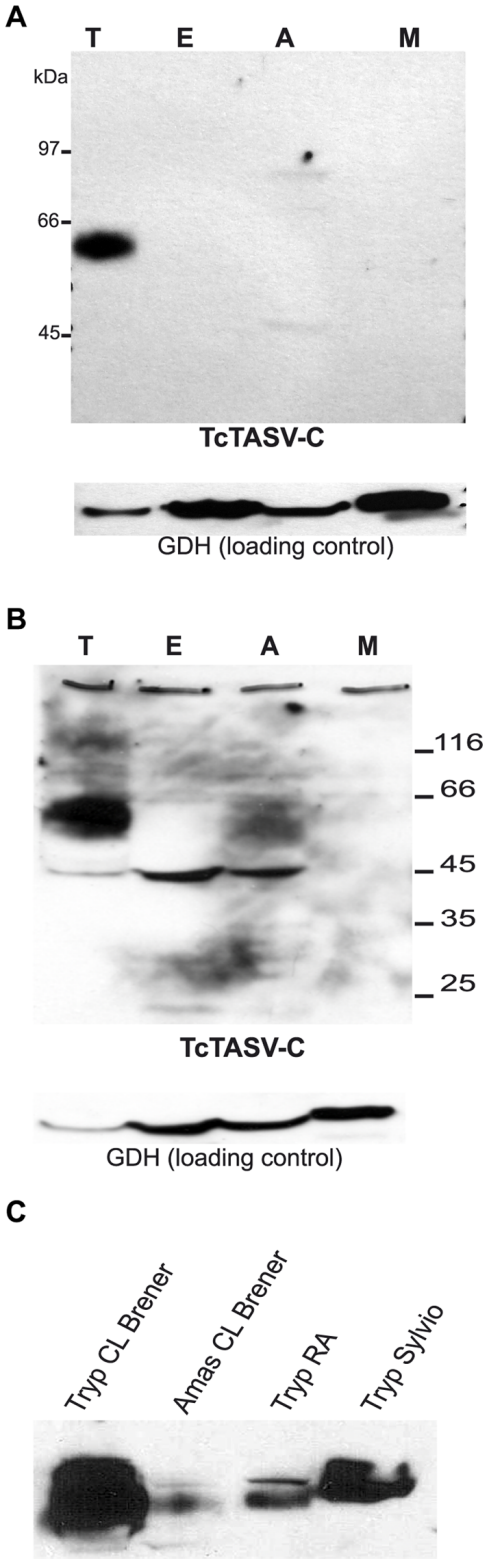
TcTASV-C subfamily is expressed mainly in trypomastigotes, and in different *T.cruzi* strains. **A.** Western blot of total protein extracts from CL Brener trypomastigotes (T), epimastigotes (E), amastigotes (A) and metacyclic trypomastigotes (M) using affinity-purified anti-TcTASV-C antibodies (upper panel). The stripped membrane was tested again with anti-GDH serum to verify comparable loading between stages (lower panel). **B.** A similar western as in (A) but over exposed to evidence the expression of TcTASV-C in other *T. cruzi* life stages**. C.** TcTASV-C expression in trypomastigotes and amastigotes from CL Brener strain and in trypomastigotes of RA and Sylvio strains.

We first analyzed the expression levels of the TcTASV-C subfamily in different stages of *T. cruzi* CL Brener strain by western blot, using purified anti-TASV-C specific antibodies (see M&M for details and File S1 for specificity of antibodies). Development of the assay showed a ∼60 kDa band in trypomastigotes, using a reagent that detects low femtograms of proteins (Fig. 2A). After a longer exposure times (O.N.) an additional band of ∼45 kDa was detected in amastigotes and epimastigotes (but not in metacyclic trypomastigotes) (Fig. 2B). As TcTASV-C genes are conserved among different *T. cruzi* isolates, we then investigated if TcTASV-C was expressed in other *T. cruzi* strains. TcTASV-C expression was detected both in RA and Sylvio, but the level of protein was variable among strains (Fig. 2C).

### TcTASV-C is attached to the parasite membrane through GPI and spontaneously shed to the medium

**Figure 3:**
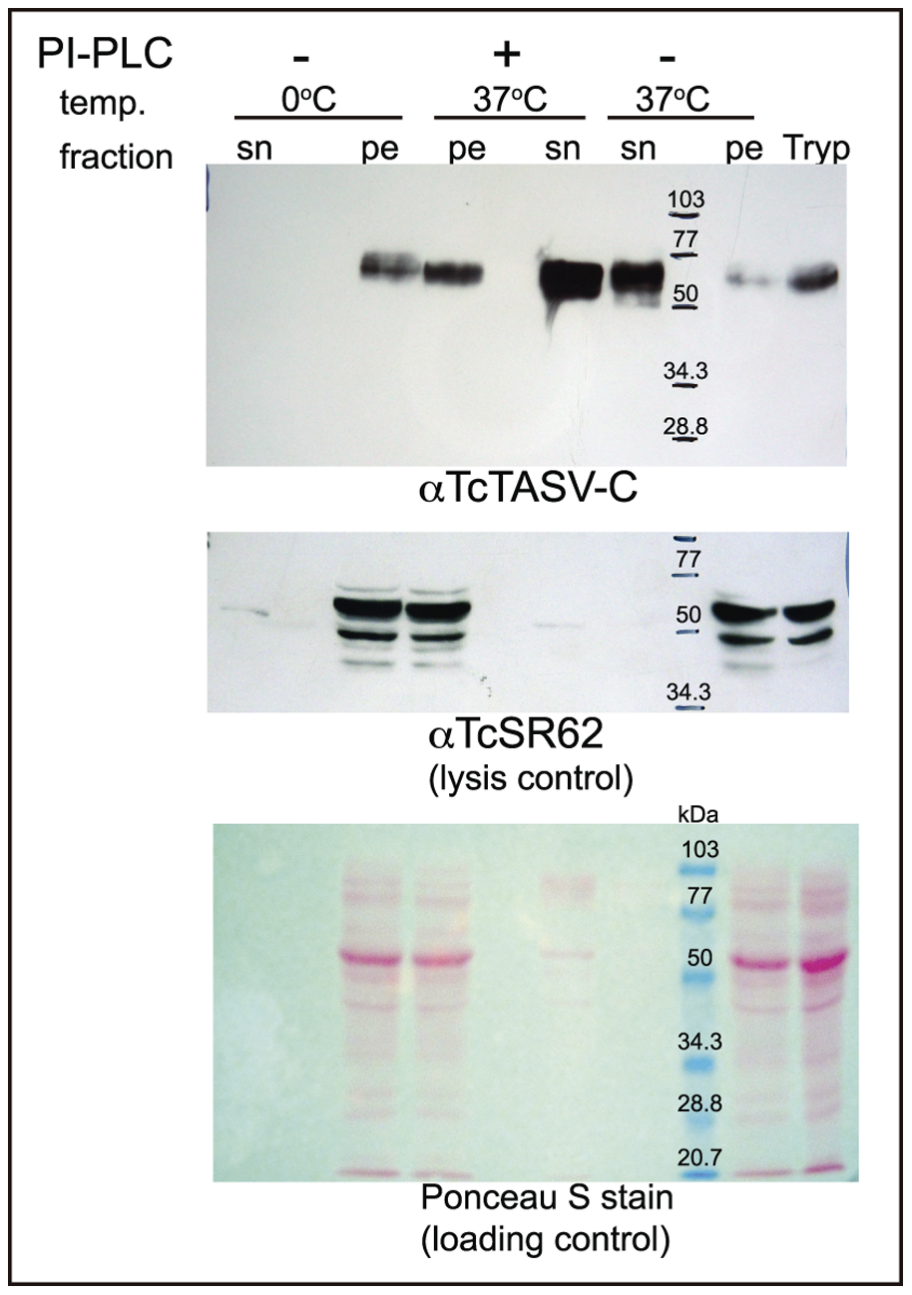
TcTASV-C is attached to the parasite membrane through a GPI anchor and spontaneously shed to the medium. Live, cell-derived CL Brener trypomastigotes were treated with 2 U of PI-PLC at 37°C, mock-treated at 37°C or left untreated at 0°C for 1 h. Parasites were centrifuged and both pellets (pe) and supernatants (sn) were analyzed by western blot using purified anti-TcTASV-C antibodies (upper panel). Trypomastigote proteins (Tryp) were also included in the western. The membrane was stripped and re-probed with anti-TcSR62 serum to verify whether there had been spontaneous lysis of the parasites (middle panel). The total protein transferred in each line is shown by Poinceau S staining (lower panel).

Given the predicted surface localization of TcTASV-C, and its potential anchoring to the membrane through a glycosylphosphatidyl inositol (GPI) anchor, we treated cell-derived trypomastigotes with Phospholipase C from *Bacillus cereus* (PI-PLC). After treatment, TcTASV-C-specific labeling was detected in supernatants, which confirms its anchoring to the membrane through GPI (Fig. 3, upper panel). In the same assay we also tested the spontaneous release of TcTASV-C into the medium. The detection of TcTASV-C in the supernatants of culture medium (Fig. 3, upper panel, PI-PLC -) indicates that this protein family is also secreted and/or shed spontaneously from the parasite surface. This process is due to an active secretion of TcTASV-C by trypomastigotes because TcTASV-C was not detected in supernatants of parasites that were incubated at 0°C. Moreover, the detection of TcTASV-C in supernatants is not due to spontaneous lysis of the parasites, as TcSR62, a constitutive cytoplasmic/nucleolar *T. cruzi* protein, could not be detected in the same supernatants (Fig. 3, middle panel).

### TcTASV-C is phosphorylated and glycosylated *in vivo*

**Figure 4:**
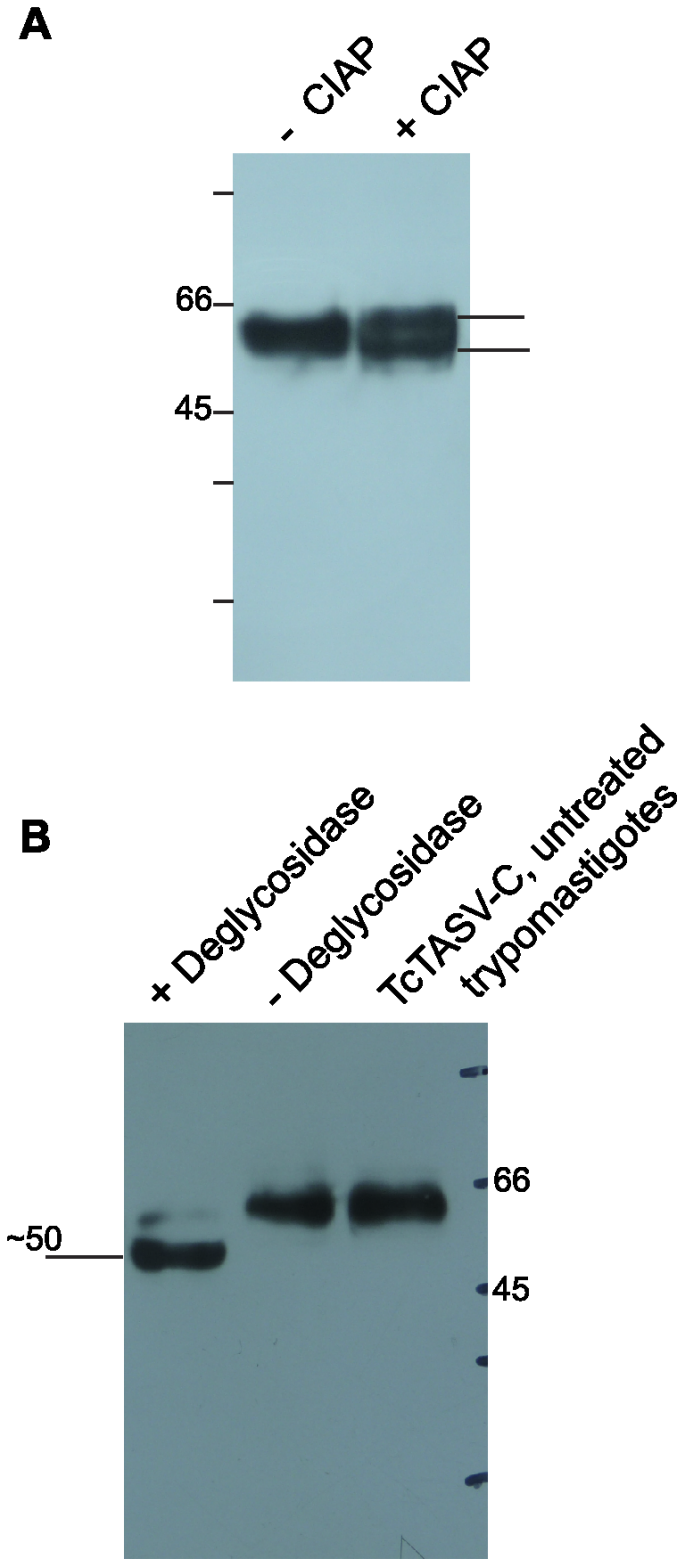
TcTASV-C is phosphorylated and glycosylated. Lysates of *T. cruzi* trypomastigotes were treated with CIAP (**A**) or glycosidases (**B**), electrophoresed on a 12% SDS-PAGE gel, transferred to nitrocellulose membrane and TcTASV-C detected using anti-TcTASV-C antibodies.

Of a total of 63 Thr, Ser and Tyr residues in the mature protein codified by *Tcruzi_1863-4-1211-93*, 33 of them were predicted to be phosphorylated (Fig. 1). Bioinformatic predictions also indicated that 19 Ser/Thr residues are putatively O-glycosylated (Fig. 1). Similar bioinformatics predictions were found for all TcTASV-C members. Besides, *Tcruzi_1863-4-1211-93* has two predicted sites for N-glycosylation (Fig. 1). These post-translational modifications could alter the migration pattern of TcTASV-C in SDS-PAGE and can help understand the differences between the expected (36 kDa) and observed (∼60 kDa) relative molecular mass of TcTASV-C (362 aa) in western blots. We therefore treated trypomastigotes either with CIAP to remove phosphates or a mixture of glycosidases to remove carbohydrates from TcTASV-C. The treatment with CIAP resulted in the migration of two bands that were detected by western blot, which indicates that TcTASV-C can be both in a phosphorylated and unphosphorylated state (Fig. 4A). On the other hand, deglycosylation of TcTASV-C produced a shift in the migration pattern of the protein of ∼10 kDa (Fig. 4B).

### TcTASV-C is clonally expressed in the surface of trypomastigotes

**Figure 5:**
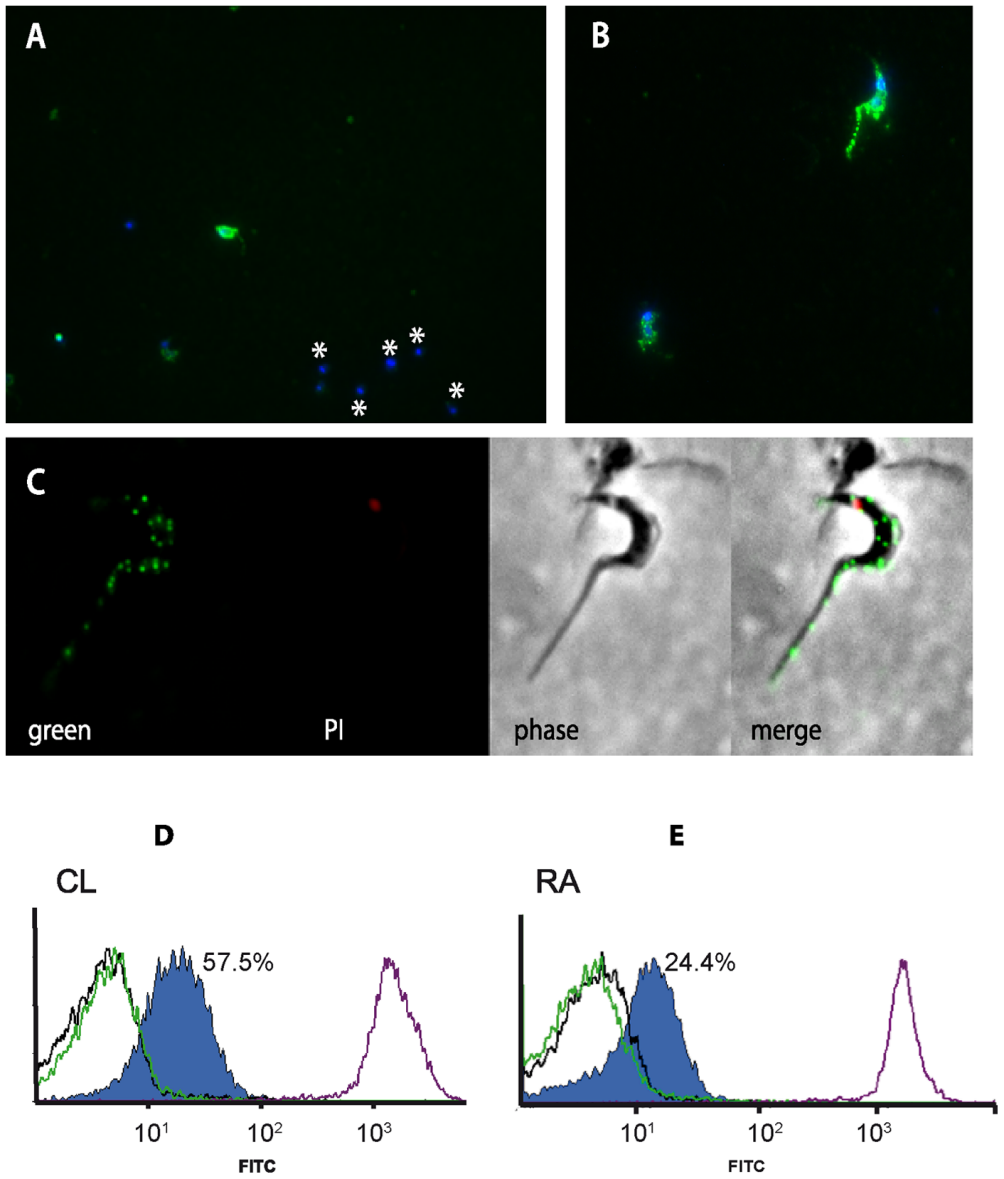
TcTASV-c is expressed at the trypomastigote surface. **A–C:** Indirect immunofluorescence was performed on unpermeabilized trypomastigotes using anti-TcTASV-C antibodies. Asterisks in A denote parasites that do not express TcTASV-C, whose DNA content was labeled with DAPI. B and C: Magnification showing the surface pattern of the TcTASV-C distribution. DNA labeling: A and B: DAPI; C: propidium iodide (PI). **D–E:** Live trypomastigotes (1×10^6^/assay) from CL Brener (D) or RA (E) strains were reacted with affinity-purified anti-TcTASV-C antibodies (blue) for 1 hour at 4°C and processed for analysis by flow cytometry. The specificity of the binding to TcTASV-C proteins was confirmed by pre-adsorption of the antibodies with the recombinant protein TcTASV-C before incubation with the parasites (pre-adsorbed, green line). Negative (IgG from normal mice; black line) and positive (sera from *T. cruzi*-infected mice; purple line) controls were included.

To determine the cellular localization of TcTASVs, trypomastigotes were labeled by immunofluorescence using anti-TcTASV-C antibodies (Fig. 5A–C). As can be observed in Fig. 5A, only a minor proportion (1 out 6 in the image shown) of the parasites were fluorescent. The labeling on positive parasites presented a particular picture of scattered dots, both on the surface of the parasite body and flagellum. This also indicates that TcTASV-C family is expressed on the parasite surface since no permeabilizing agent was used (Fig. 5B, C).

To verify the predicted location of TcTASV-C and immunoflourescence results, we labeled live RA and CL Brener trypomastigotes with anti-TcTASV-C antibodies, and then analyzed them by flow cytometry. As shown in Figure 5D–E, TcTASV-C was detected on the surface of trypomastigotes in both *T. cruzi* strains. Only 57.5% (CL Brener, Fig. 5D) and 24.4% (RA, Fig. 5E) of the parasites were FITC positive, which indicates that not every trypomastigote expresses TcTASV-C on their surface at the same time. These results also show that TcTASV-C is expressed in a greater number of cells in CL Brener than in RA strain, which is in agreement with our previous western blot results. Besides, most positive events showed moderate fluorescence intensity, indicating that TcTASV-C is expressed at a moderate level. To confirm the specificity of binding, TcTASV-C antibodies that had been previously pre-adsorbed with the recombinant protein TcTASV-C_GST_ were also incubated with trypomastigotes, in which case no differences were observed with the labeling obtained by pre-immune antibodies (Fig. 5D–E, green line: pre-adsorbed; black line: IgG from control mice).

### TcTASV-C is recognized by sera of infected hosts

**Figure 6:**
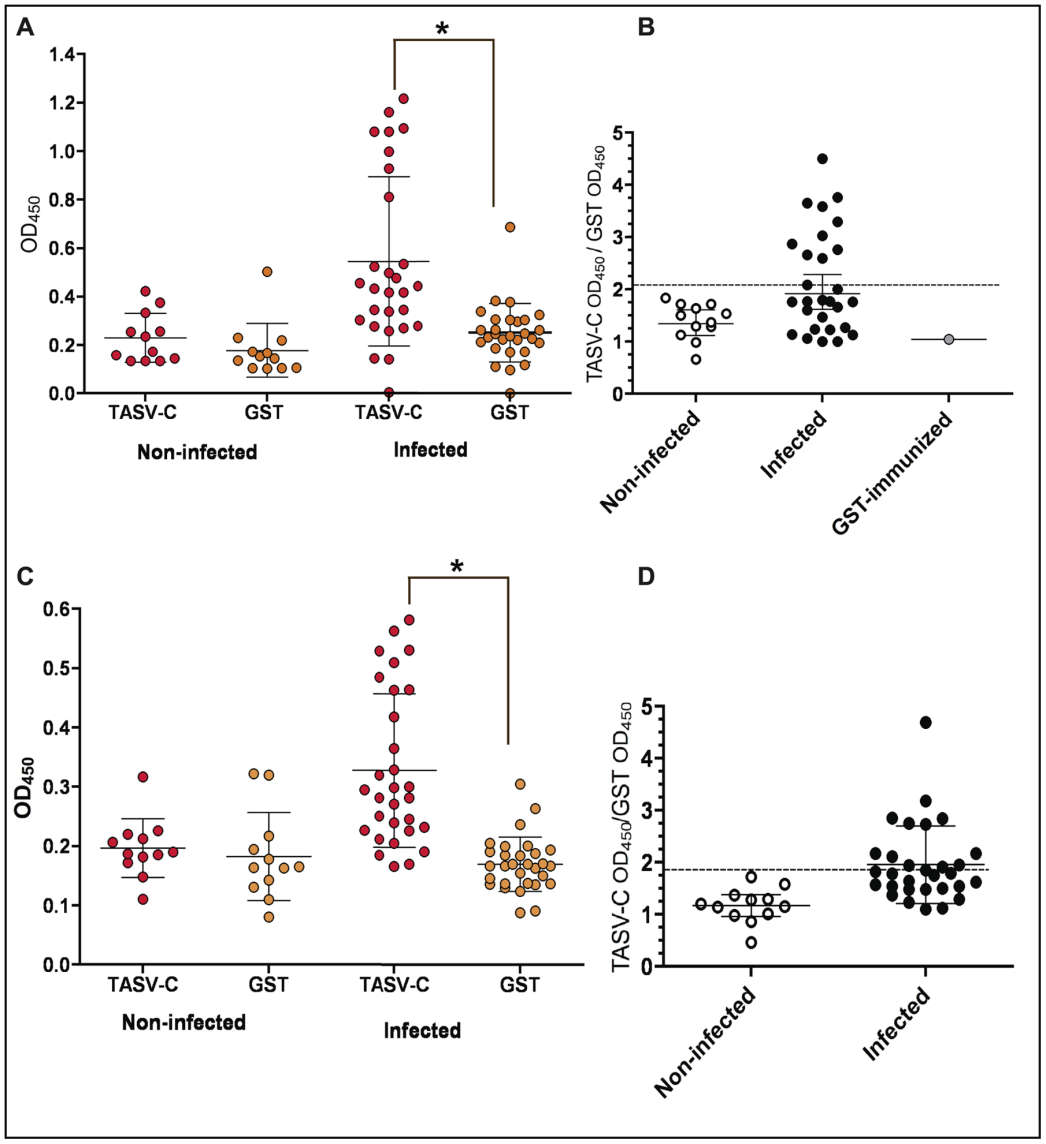
TcTASV-C is recognized by sera from infected hosts. The reactivity of sera from rabbits (A, B) and humans (C, D) against TcTASV-C (and GST) was evaluated by ELISA. Results are presented as absorbance at 450 nm (A, C) or as the ratio between the ODs obtained for each serum against TcTASV-C and GST (B, D). Asterisks in A and C denote differences in the mean values (p<0.005). Dotted lines in B and D represent the cut-off, calculated as the mean + 2SD of control (uninfected) sera. Human's sera: infected: N = 30; uninfected: N = 12. Rabbit's sera: infected: 28; uninfected: 12.

To further characterize TcTASV-C, we evaluated its capacity to induce specific antibodies in *T. cruzi* infected hosts. Sera from rabbits infected with different *T. cruzi* lineages were assayed by ELISA to evaluate their reactivity against TcTASV-C_GST_. The mean reactivity -measured as the average of optical densities (ODs) of sera against TcTASV-C_GST_- was higher in infected than in non-infected animals (infected: 0.5579±0.35; non-infected: 0.2298±0.10; p = 0.0005). The average reactivity of the same group of sera against GST, the protein used as background control, was similar in both groups (infected: 0.2419±0.13; non-infected: 0.1784±0.11; p = n.s.) (Fig. 6A). These differences in the mean values did not reflect the fact that –among *T. cruzi* infected rabbits- some sera were reactive to TcTASV-C and some others were not. To distinguish which individual sera were those specifically reacting with TcTASV-C, we calculated the ratio of the OD values obtained for each serum against TASV-C_GST_ and GST (Fig. 6B). Ten out of 28 sera (35.71%) of infected rabbits reacted with TcTASV-C. However, with these serum samples we could not see any correlation between reactivity to TcTASV-C and lineage of the *T. cruzi* infecting strain (File S3). In a murine model of *T. cruzi* infection, anti-TcTASV-C antibodies were detected starting from 20 days post-infection. This finding was coincident with the peak of circulating trypomastigotes, and the acute phase of infection (File S4). We also evaluated the reactivity of a panel of 42 human sera (30 positive and 12 negative, as indicated by anti-*T. cruzi* serology) provided by the serum bank of the Instituto Nacional de Parasitología “Dr. Mario Fatala Chaben” (Ministerio de Salud, ANLIS, Buenos Aires, Argentina). As observed for rabbit's sera, the mean reactivity of infected people against TcTASV-C_GST_ was higher than controls (infected: 0.3280±0.13; non-infected: 0.1966±0.05; p = 0.0005), while no differences were found in the reactivity against GST between both groups (Fig. 6C). Ten sera from infected individuals (33%) were reactive to TcTASV-C, as calculated by the TcTASV-C/GST ratio (Fig. 6D). This result is particularly relevant because it indicates that TcTASV-C is antigenic in the natural infection, not only in an experimental model of the disease but also in the natural cell cycle involving humans.

## Discussion

Most of the works about multigene families in *T. cruzi* that were carried out before the completion of the *T. cruzi* genome studied highly expressed proteins (mucins, trans-sialidase, cruzipain, amastin, amongst others) [13], [18], [49], [50]. On the other hand, the multigene family MASP was discovered when the parasite's genome was sequenced and annotated, and is a good example of how a genome project shed light into the structure of a genome [11], [17], [51]. Although the MASP family is composed by a very large number of genes (>1400), the delay in its identification was probably due to its unusual or not-so-abundant protein expression pattern. Therefore, it is no surprise that the TcTASV gene family –composed by a much lower number of genes- was not noticed even after the completion of the sequencing of the *T. cruzi* genome.

Several years ago, we started a project to identify genes expressed preferentially in the different stages of *T. cruzi* by means of ESTs sequencing [34], [52], [53]. The analysis of an epimastigote-substracted trypomastigote cDNA library led us to the identification of a novel gene family, named TcTASV [34]. In the early versions of the *T. cruzi* database (TcruziDB and TriTrypDB 1.0–2.3) some TcTASVs genes were annotated as hypothetical or mucin-like genes, while others were solely marked as open reading frames [54]. In our previous work we demonstrated that TcTASV was indeed a novel gene family, conserved among different *T. cruzi* lineages and with no orthologs in other species (including trypanosomatids). Added to these characteristics, the TcTASV family turns out to be an attractive target for study since a TcTASV-C gene was identified among a pool of protective vaccine antigens [37].

Here we demonstrate that TcTASV-C proteins are expressed in *Trypanosoma cruzi*, mainly in the trypomastigote stage. We also found that TcTASV-C is localized at the cellular surface, anchored to the membrane through a GPI moiety and released spontaneously to the milieu. We also determined that TcTASV-C is not a major component of the parasite surface. The detection of TcTASV-C by western blot was achieved by using a reagent that detects low femtograms of protein in the equivalent of ∼20×10^6^ trypomastigotes. This was also supported by flow cytometry and immunofluorescence experiments, where few labeled parasites were observed among the whole population. Both western blot and flow cytometry data also showed that the expression of TcTASV-C is variable among different *T. cruzi* strains, being much more abundant in CL Brener than RA, despite the fact that both strains belong to the same lineage. In *T. brucei*, several minor components of the cell surface turned out to have important functions in maturation of infection [55], [56]. In that sense, Fragoso *et al*. (2009) [55] demonstrated that the phosphoprotein PSSA-2 is essential to colonize the salivary glands of the tse-tse fly and to produce metacyclic forms. Moreover, the correct localization and function of this protein in the plasma membrane is dependent on the phosphorylation of a cytoplasmic residue of threonine.

We have also demonstrated here that TcTASV-C expression follows a clonal expression pattern, *i.e.* in a certain parasite population the expression of this subfamily is not uniform. This is particularly interesting since it opens an additional question about the regulation mechanism of expression of surface proteins in *T. cruzi*. Protein translation in trypanosomatids is tightly regulated by the interaction of cis-acting elements (mainly the 3′ UTR of the genes) and RNA binding proteins [57]. In the case of the TcTASV-C family, the 3′UTR is highly conserved, and is actually a ‘hallmark” of the gene family. A similar situation has been recently described for masp genes [51]; in their work, dos Santos *et al*. (2012) suggested that subtle nucleotide differences in the 3′UTR regions can alter the interaction with regulatory proteins that favor or prevent the translation of specific transcripts. However, this hypothesis does not explain why the expression of a protein (in our case TcTASV-C) is silenced in some parasites but active in others. Recent findings indicate the existence of the base J and epigenetic regulation of gene expression in *T. cruzi* [58], [59]. In *T. brucei*, epigenetic modifications are involved in the control of antigenic variation [60], [61], which suggest this kind of regulation for the TcTASV family as a possibility to be studied.

Another interesting characteristic of the expression pattern of TcTASV-C is its distribution on the parasite membrane as dots (patches) that resemble detergent resistant membrane domains (DRMs). Some proteins that are phosphorylated and anchored to the plasma membrane by GPI can be localized in lipid rafts. This has been demonstrated in *T. brucei* for PARB, which is present in small discrete spots distributed over the entire cellular surface [62]. TcTASV-C presents these characteristics, and even though we did not assay if TcTASV-C is associated to DRMs, this could be a possibility. Nevertheless and as mentioned above, the phosphorylation of a protein can alter its behavior in a wide range from function, signal transduction or subcellular localization, among others. In *T. brucei*, the differential phosphorylation state of the procyclins EP and GPEET (two GPI-anchored proteins) is coordinated and changes through the life cycle of the parasite. Indeed, the phosphorylation/dephosphorylation state has been linked to the membrane localization (flagellar pocket *vs.* cell surface) [63]. In our case, we have detected the expression of TcTASV-C by western blot both in trypomastigotes and, although in a much lower level, in epimastigotes and amastigotes. However, and in contrast to findings in trypomastigotes, we failed to detect the expression of TcTASV-C at the cellular surface in non-permeabilized epimastigotes (data not shown). This could reflect a differential phosphorylation state in TcTASV-C in both parasite stages that might regulate its localization and eventually its activity.

Although we have found that the TcTASV-C subfamily is expressed in trypomastigotes, no TcTASV-C peptides were identified in the trypomastigote proteome [36]. We also determined here that TcTASV-C is glycosylated and is anchored to the membrane through a GPI anchor. In the work of Nakayasu *et al* (2012), trypomastigotes were lysed by sonication, which does not favor the extraction of membrane-bound proteins. Moreover, some highly glycosylated proteins (like TcTASV-C) cannot be digested correctly by standard methodologies, which also can explain its non-detection in MS-MS analysis. On the other hand, 5 TcTASV-A genes were identified in the trypomastigote proteome, in agreement to our evidences of the intracellular localization of TcTASV-A in trypomastigotes (unpublished observations). The proteomes of metacyclic trypomastigotes and epimastigotes –designed to preferentially identify membrane-bound and/or hydrophobic proteins- failed to find any TcTASV peptide [64], in line with results presented here that showed a minimum expression (or absence) of TcTASV-C in insect-derived parasite-stages.

The reactivity to the recombinant TcTASV-C displayed by sera from animals and humans infected with *T. cruzi* demonstrated the *in vivo* expression of TcTASV-C and its contact with the immune system of the host. Furthermore, the heterogeneous reactivity (detected in ∼30% of sera both in natural and experimental infections) remained elusive any possible association with the infecting strain or status of the infection (*i.e.* acute vs chronic), and also reflects the complex humoral response elicited by *T. cruzi* infection. Future studies will be necessary to determine if TcTASV-C reactivity can be a predictor of disease evolution, since the percentage of reactivity observed is quite similar to those of the patients that develop symptoms at the chronic phase of the disease.

In brief, we have demonstrated here that TcTASV-C is a novel protein family in *T. cruzi* expressed on the surface of trypomastigotes. TcTASV-C is phosphorylated, heavily glycosylated, shed to the medium and is in contact with the immune system of the host during the course of the natural infection. All these characteristics are particularly interesting since the trypomastigote is the parasite stage that circulates in blood and disseminates the infection to secondary organs by invasion of novel cells. Future work is needed to determine the function of TcTASV-C, but our current hypotheses include both its putative involvement in immune evasion and host-parasite interactions.

## Supporting Information

File S1 **Specificity analysis of anti-TcTASV-C antibodies by competition assays.** Total protein extracts from CL Brener trypomastigotes (T), epimastigotes (E) and amastigotes (A) were electrophoresed on 10% acrylamide gels and transferred onto nitrocellulose membranes. After blocking with PBS containing 3% non-fat milk, membranes were probed with affinity-purified anti-TcTASV-C antibodies that had been pre-adsorbed with recombinant TcTASV-C_GST_ (left panel), recombinant TcTASV-B_GST_ (middle panel) or left untreated (right panel). IgG pre-adsorption was carried out by incubating the antibody solution with the recombinant proteins at 0.5 µg/ml for 1 h at 4°C. Development was carried out as indicated in the Materials and Methods section. The stripped membrane was tested again with anti-GDH serum to verify comparable loading between stages (lower panel).
(JPG)

File S2 **Genes and ORFs of the TcTASV family.** The TriTrypDB was searched to identify all the genes and open reading frames (ORFs) from CL Brener strain that belong to the TcTASV family. The subfamilies are clearly indicated.
(XLS)

File S3 **Reactivity against TcTASV-C is not associated with** ***T. cruzi*** **infecting strain.** The reactivity of individual sera from *T. cruzi* infected rabbits is plotted showing the *T. cruzi* strain that infected each animal.
(PDF)

File S4 **Follow up of parasitemia and anti-TcTASV-C antibodies in an experimental murine model of** ***T. cruzi*** **infection.** Mice (n = 4) were infected with 100 trypomastigotes of the RA strain (TcVI). The levels of circulating parasites and anti-TcTASV-C antibodies (ELISA) were monitored during the course of infection. The graphs show parasitemia (trypomastigotes/ml) and anti-TcTASV-C reactivity (OD at 450 nm), both expressed as mean ± SD (upper panel) and the anti-TcTASV-C reactivity of the individual mice during the course of infection (lower panel).
(PDF)
