# Supplementary figures and images for: TcTASV-C, a Protein Family in Trypanosoma cruzi that Is Predominantly Trypomastigote-Stage Specific and Secreted to the Medium
Source: PLoS One. 2013 Jul 29;8(7):e71192. doi: 10.1371/journal.pone.0071192 (PMC3726618; doi:10.1371/journal.pone.0071192)

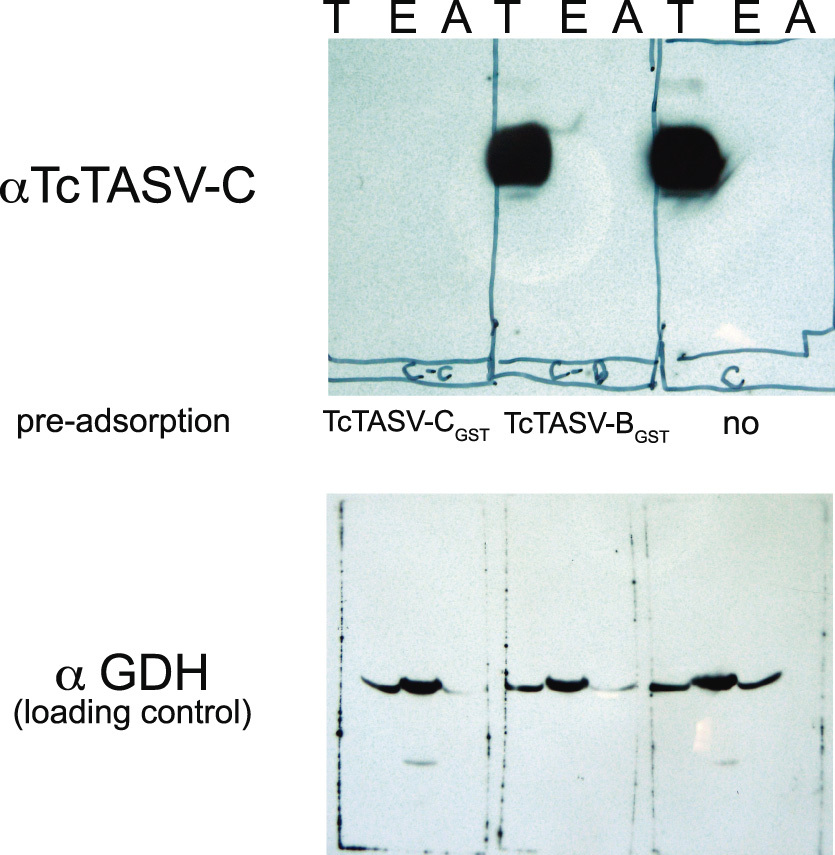

Supplement: File S1 — Specificity analysis of anti-TcTASV-C antibodies by competition assays. Total protein extracts from CL Brener trypomastigotes (T), epimastigotes (E) and amastigotes (A) were electrophoresed on 10% acrylamide gels and transferred onto nitrocellulose membranes. After blocking with PBS containing 3% non-fat milk, membranes were probed with affinity-purified anti-TcTASV-C antibodies that had been pre-adsorbed with recombinant TcTASV-CGST (left panel), recombinant TcTASV-BGST (middle panel) or left untreated (right panel). IgG pre-adsorption was carried out by incubating the antibody solution with the recombinant proteins at 0.5 µg/ml for 1 h at 4°C. Development was carried out as indicated in the Materials and Methods section. The stripped membrane was tested again with anti-GDH serum to verify comparable loading between stages (lower panel). (JPG) [file pone.0071192.s001.jpg]

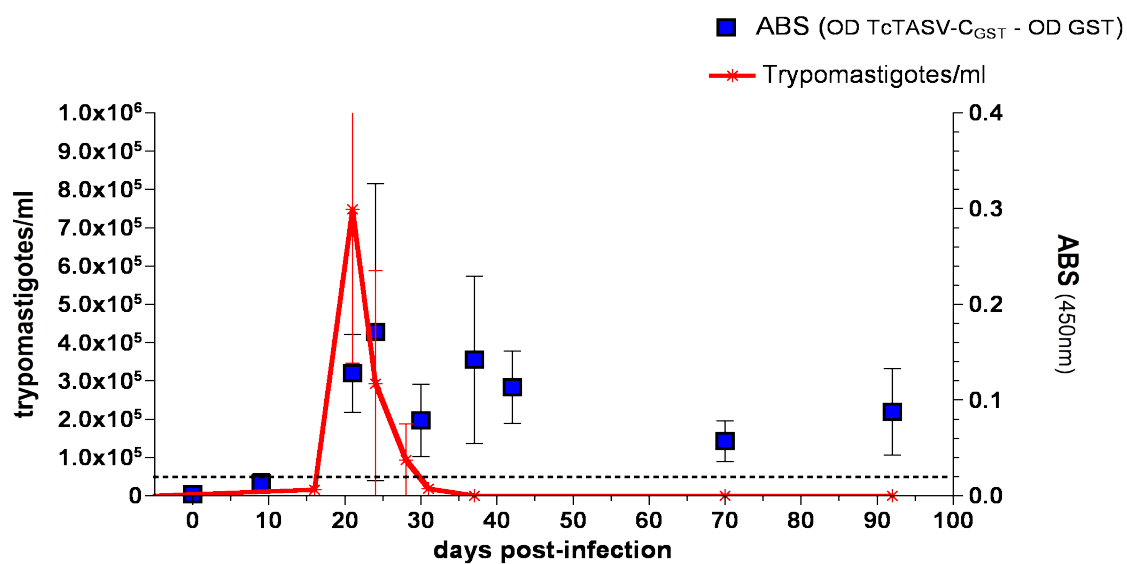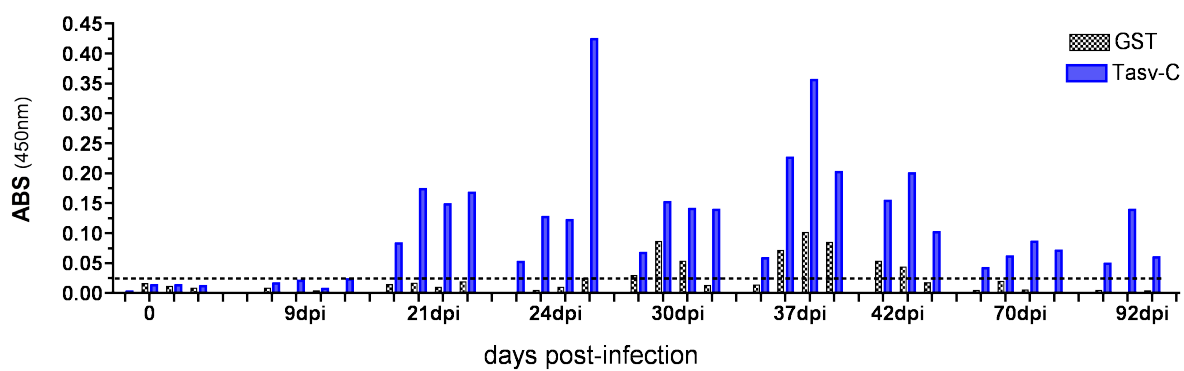

Supplement: File S4 — Follow up of parasitemia and anti-TcTASV-C antibodies in an experimental murine model of T. cruzi infection. Mice (n = 4) were infected with 100 trypomastigotes of the RA strain (TcVI). The levels of circulating parasites and anti-TcTASV-C antibodies (ELISA) were monitored during the course of infection. The graphs show parasitemia (trypomastigotes/ml) and anti-TcTASV-C reactivity (OD at 450 nm), both expressed as mean ± SD (upper panel) and the anti-TcTASV-C reactivity of the individual mice during the course of infection (lower panel). (PDF) [file pone.0071192.s004.pdf]
