# Supplementary material for: TcTASV-C, a Protein Family in Trypanosoma cruzi that Is Predominantly Trypomastigote-Stage Specific and Secreted to the Medium
Source: PLoS One. 2013 Jul 29;8(7):e71192. doi: 10.1371/journal.pone.0071192 (PMC3726618; doi:10.1371/journal.pone.0071192)
Supplement: File S3 — Reactivity against TcTASV-C is not associated with T. cruzi infecting strain. The reactivity of individual sera from T. cruzi infected rabbits is plotted showing the T. cruzi strain that infected each animal. (PDF) [file pone.0071192.s003.pdf]

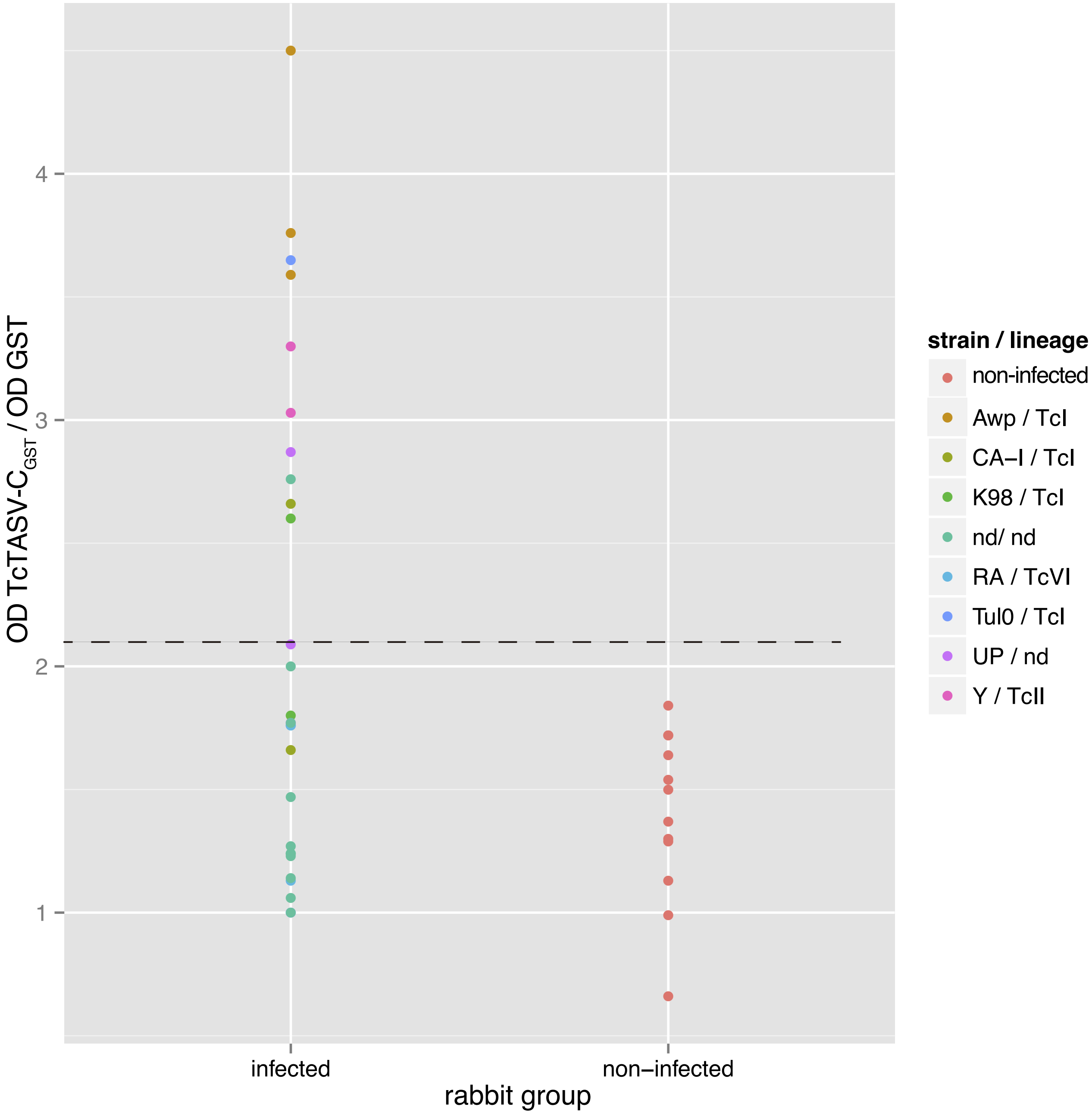
OD TcTASV-C$_{GST}$ / OD GST
1
2
3
4
infected
non–infected
rabbit group
strain / lineage
non-infected
Awp / TcI
CA–I / TcI
K98 / TcI
nd/ nd
RA / TcVI
Tul0 / TcI
UP / nd
Y / TcII
